# Retraction: Propofol Prevents Autophagic Cell Death following Oxygen and Glucose Deprivation in PC12 Cells and Cerebral Ischemia-Reperfusion Injury in Rats

**DOI:** 10.1371/journal.pone.0275548

**Published:** 2022-09-28

**Authors:** The PLOS ONE Editors

Following the publication of this article [1], concerns were raised regarding text overlap with previously published work and results presented in multiple figures. Specifically,

- Overlap was identified between text in the Results and figure legends of this article [1] and text in an article that was published previously [2] and does not appear to have been cited appropriately in [1].
- The following Western blot loading control panels appear similar despite representing different experimental conditions:
  - Lanes 1–4 of the GAPDH panel in Figure 2A, and the GAPDH panels in Figures 7E, 12B and 12E.
  - Lanes 2–5 of the GAPDH panel in Figure 2E and the GAPDH panel in Figure 7A.
  - Lanes 4–7 of the GAPDH panel in Figure 5A and the GAPDH panel in Figure 7B.
  - The GAPDH panel in Figure 2F and the GAPDH panel in Figure 3A.
- In Figure 8 of this article [1] and Figure 7A of an article published later [3], the following panels appear to partially overlap when rotated or flipped:
  - Figure 8A in [1] and the CA-PFT CA1 panel in Figure 7A of [3].
  - Figure 8B in [1] and the CA+3-MA CA1 panel in Figure 7A of [3].
  - Figure 8C in [1] and the CA+3-MA CA3 panel in Figure 7A of [3].
  - Figure 8E in [1] and the CA+DMSO CA1 panel in Figure 7A and the CA+DMSO CA1 and CA+3-MA (3h after ROSC) CA3 panels in Figure 7C of [3].
  - Figure 8F in [1] and the CA+DMSO CA3 panel in Figure 7C of [3].
  - Figure 8G in [1], and the zoomed-out panels for CA+BDA in Figure 7A and CA+3-MA panels in Figure 7C of [3].

The corresponding author stated that overlapping text between the articles [1,2] may have occurred due to similarities in the topics and methods described. Given the extent of text overlap, the editors consider that this article does not adhere to ethical publishing standards.

The corresponding author noted that several western blot panels may have been re-used in error during the preparation of Figures 3, 5, 7 and 12. They provided replacement images and files described as raw image data underlying the western blots. Given the number of issues across several figures, the editors remain concerned about the reliability of the published results and replacement data.

In relation to the re-use of images from this article [1] in a later article [3], the corresponding author stated that data were stored incorrectly which led to accidental re-use in the later article [3]. The editors remain concerned about the reliability of the results given the errors in data handling.

In light of the overlapping text and concerns affecting multiple figures that question the reliability of these data, the *PLOS ONE* Editors retract this article.

DC and WJ did not agree with the retraction, stand by the article’s findings, and apologize for the issues with the published article. LW, AQ, QZ, and XZ either did not respond directly or could not be reached.
